# Experiences of nurses providing maternity care in a public hospital during the COVID-19 pandemic in Nepal: A qualitative study

**DOI:** 10.1371/journal.pgph.0000322

**Published:** 2022-05-05

**Authors:** Bidhya Basnet, Pratiksha Chapagain, Sabitra Subedi, Tulasha Dahal, Saraswati Neupane, Ranjita Khanal, Richard J. Pinder, Don Eliseo Lucero-Prisno, Shyam Sundar Budhathoki

**Affiliations:** 1 Maternal Health Nursing Department, Biratnagar Nursing Campus, Institute of Medicine, Tribhuvan University, Biratnagar, Nepal; 2 Department of Primary Care and Public Health, School of Public Health, Imperial College London, London, United Kingdom; 3 Department of Global Health and Development, London School of Hygiene & Tropical Medicine, London, United Kingdom; Jhpiego, UNITED STATES

## Abstract

Maternity service providers have struggled to provide high-quality services to women and newborns during the ongoing COVID-19 pandemic which has substantially impacted health systems and disrupted maternity services globally. Nepal is a resources-limited country that reported a significant impact of the pandemic on maternal health services. It is therefore important to understand better the perspective of health care professionals in this context. This study intends to explore the experiences of nurses providing maternity care in the public sector during the COVID-19 pandemic in Nepal. A qualitative study using a phenomenological design was conducted. Altogether ten nurses working in maternity services were selected using purposive sampling technique. Data were collected by face-to-face in-depth interviews using a semi-structured interview guide. Thematic analysis was conducted using Clarke and Braun 2006 technique. The findings of the study were organized into codes, sub-themes and themes. The six themes identified were fear of COVID-19 at work, challenges at work, changes at work and services, motivations to work, stigma due to COVID-19, and impact on services. Participants described how maternity services could not be stopped during the pandemic. They had experienced decreased utilization of antenatal services as a consequence of ‘lockdown’ thereby leading to an increase in maternal and neonatal mortality. Respondents reported ineffective human resource management compromising the quality of care. The professional responsibility to cope with adverse circumstances and serve society is a major source of motivation that health workers relied upon to get them through the pandemic period. A wide range of challenges were faced by service providers during the pandemic which requires action and support of all levels of government, institutions and society-at-large to assure the continued provision of safe maternity care during such a protracted period of challenging work.

## Introduction

The novel coronavirus (SARS-CoV-2) was first identified in Wuhan, China in late 2019 and was declared a pandemic by the World Health Organization (WHO) in March 2020. Intensive efforts are ongoing worldwide to establish effective treatment, curb disease transmission and roll out vaccination [1–3]. In Nepal, the first case was observed on 13^th^ January 2020 and the number of cases has remained on an upward trajectory [4]. Various measures have been adopted to minimize human to human transmissions including travel restrictions, quarantine, and stay at home orders, affecting the population’s ability to access health services [5].

Before the COVID-19 pandemic, globally 810 women died each day due to preventable causes related to pregnancy and childbirth [6]. The COVID-19 pandemic poses considerable challenges for countries to maintain high quality, essential maternal and newborn health services [7]. Moreover, the anatomical, physiological and immune status changes during pregnancy confer additional health risks, leading to maternal morbidity sometimes requiring intensive care unit (ICU) admission and potentially precipitating perinatal death [8]. Countries around the world reallocated significant resources, affecting all health services including maternity services switching from regular services to emergency response. Access to maternity services is further complicated by travel restrictions, transport disruption and community-level fear of transmission. The transition in maternal health care provision from traditional face-to-face consultations towards alternative modalities remote consultations has constrained the support for pregnant women, with consequent adverse impacts on pregnancy experience and outcomes [9]. Various studies and sources have shown reductions in utilization of maternity services, mounting evidence of over-medicalization, and an increase in maternal mortality and morbidity [10–17]. It has been estimated that even with a modest decline of 10% in coverage of maternity and newborn health care, an additional 28,000 maternal deaths and 168,000 newborn deaths in low and middle-income countries could occur annually [18].

During public health emergencies, health care workers and their roles are vital in disease control. Mobilizing health care providers during a crisis is always a challenge. Studies carried out during past outbreaks have demonstrated the heightened risk of disease transmission among health workers [19] as well as additional stress attributed to stigmatization, understaffing, and uncertainty [20]. As the world battles COVID-19, pregnancies continue and babies are born, meaning the continuity of effective maternity health provision remains vital. Therefore providers must adapt to a rapidly changing environment. Maternity providers may experience stress from a range of sources whilst working during the outbreak [5]. Shortages of qualified staff along with an increased level of stress due to workload compounded with fear of nosocomial infections among the maternity care providers owing to the lack of personal protective equipment are reported [21]. Previous studies have also shown that the care providers’ personal, professional lives and their mental health have been severely affected during pandemic situations [22,23]. Nepal reported a severe impact on maternal and neonatal care due to the COVID-19 pandemic with a decrease in institutional delivery by half compared to the pre-pandemic data [17,24]. This calls for inquiry on the experiences of the maternal care providers to understand the potential challenges of delivering services during the pandemic including the service providers’ physical, emotional and societal influences on their practice in Nepal. There is considerable peer-reviewed literature reporting experiences of health workers in general from across income settings [25,26] but more limited reporting in maternity care providers [27]. Accordingly, a deeper exploration of the pandemic’s impact in a low resource maternity setting is of value. This study aims to explore the experiences of maternity service providers during the pandemic, examining their perspectives from the point of individuals, families, society, institutions and government. The findings of the study will provide an opportunity to learn about impact, and identify strategies to assure the provision of maternity care going forward.

## Materials and methods

The 32-item checklist of consolidated criteria for reporting qualitative research (COREQ) was used to report the methodology and result section of this study [28].

### Ethics and consent to participate

This study was approved by the Institutional Review Committee of Tribhuvan University, Institute of Medicine, Kathmandu (Ref: 55(6–11)E^2^077/078). The potential participants were contacted over the telephone. Of those who expressed an interest, an in-person visit was made to provide the written information sheet. With adequate time given for understanding and clarifying the information sheet, the participants who agreed to participate signed the informed consent form before the interview was conducted. The provision for voluntary participation ensured that participants could withdraw anytime from the study up to the point of analysis. Confidentiality was maintained throughout the study by using numerical identifiers on interview notes, audio recordings and transcripts.

### Research team

This study was conducted by a team of nine. The first six authors are female and maternal health faculty trained in qualitative research. These six members of the research team, as nursing educators have a professional relationship with most of the participants as placement providers for the student nurses sent by the nursing educators. This relationship played a constructive role in conducting this research in terms of rapport building between researcher and participant. The relationship that existed prior was strictly professional as staffs of placement provider and education institute. Whereas the last three authors of this study are male medical doctors with experience in public health and health systems and have experience of conducting qualitative studies. The first author and third author conducted the interviews.

### Study design

The phenomenological approach of qualitative study design was used as this approach helped in exploring personal perspectives from nurses working in maternity care during the pandemic. Qualitative data were collected using in-depth interviews using a semi-structured interview schedule.

### Participant selection

All maternity service providers who were working in front-line health care providers were eligible for the study. We selected maternity nurses as our study population as they were involved in both antenatal care as well as inpatient maternity care including delivery and postnatal care. To date in Nepal, regular maternity services are all provided by nurses who have midwifery skills and training. Nurses working in midwifery services do not have or need a separate midwifery licence. In Nepal, midwives as separate professionals is an emerging concept, as courses training midwives have only started in recent years [29].

Participants were recruited through purposive sampling and special consideration was given to the different elements of the maternity department and COVID-19 history of the providers. Participants with different characteristics linked with the roles they take up in the maternity department. Participants have roles as managers, unit-in charges and floor nurses. The sample size was determined by data saturation. In-depth interviews were conducted among seven floor nurses and three unit in-charges. Floor nurses in our context are registered nurses who are primarily involved in providing one-to-one care to the patients.

### Study setting

This qualitative study was conducted in a Koshi Hospital, Biratnagar. Biratnagar is the largest city of Province One with a population of 242,548 and is located in the Terai belt [30]. Koshi Hospital was previously a zonal hospital but as Nepal entered into the three-tier government system, Koshi Hospital, located and operating in Province One, is now under the purview of the central government and serves as a tertiary level referral hospital. It has 350 beds and 17 departments. It provides preventive, promotive and curative services to the public. It is the main referral hospital for maternity services in Province One and has a capacity of 100 maternity beds. It provides maternity services through a safe motherhood program. Services are delivered through the antenatal ward, labour room, post-operative ward, postnatal ward, Maternal and Child Health (MCH) clinic and outpatient department. Being a public hospital, it delivers maternity services under the government’s safe motherhood program. Under this program, women can undergo delivery free of cost and also receive incentives based on their residence. Women receive maternity services through the outpatient department and maternal health clinic. Upon admission, women are received into the admissions room, whereafter they may elect to remain within the free-to-access health system or move to a ‘cabin’ that requires payment. Paying for this upgrade renders the woman ineligible for the state-funded safe motherhood program which covers medical supplies and hospital fees. As nurse-patient ratios are a challenge to maintain in most public hospitals in Nepal, ad hoc and informal non-clinician carers (including family members) provide support on the ward. A typical patient can have upto five non-clinical carers also called as ‘attendants’ supporting them. While visitors (including husbands) are not allowed into delivery rooms or operating theatres during delivery, these visitors are responsible for comforting the patients, fetching medicines from pharmacies, arranging food and clothes for the patient and at times assisting with patient mobility, transporting samples to the lab and fetching reports.

### Data collection

Data were collected using a semi-structured interview guide. A semi-structured interview guide was developed based on previous relevant literature, the research question and informally interviewing four maternity service providers in a similar setting. The interview guide was reviewed by the research team and translated to the local language. The translated guide was further validated by a bilingual expert from within the research team. An in-depth interview was conducted using local language in their own work setting. The in-depth interviews lasted between 40–80 minutes as a single session. n Data were collected over a period of two months (September to November 2020). In-person interviews were carried out using personal protective measures at their workplace. Probes were used in the interview to achieve saturation of the information from the interview [31].

### Data analysis

Thematic analysis was conducted using the Clark and Braun 2006 [32], deductive/inductive approach. The analysis was conducted in six stages; familiarizing with the data, generating initial codes, searching themes, reviewing themes, defining and naming themes and writing up the findings. The verbatim dialogue was transcribed in the local language. Two researchers primarily conducted the transcription and translation. The data were anonymised at the stage of transcription itself where all identifiable information was removed. The translation was verified by a fluent bilingual researcher and a native English language speaker both within the research team.

After familiarization with the content of the transcript, all the relevant pieces of data related to the research question were manually identified as codes and data relevant to each code were further collated. The list of codes was prepared at this phase, which was later sorted into potential themes. A table was formulated and codes were further organized into each theme. The codes, sub-themes and themes relevant to the research question were formulated. The relationship between codes, themes, and different levels of themes was explored further. The themes were further analyzed where some themes were collapsed and others separated into discrete themes. Member checking was done with two participants using the synthesized analysed data to ensure rigorness of data analysis [33]. The relationship of themes with data was explored to establish the validity of each theme. In this phase, the importance of each theme (and associated data) was determined. The dialogue that best captured the theme was identified to proceed into the other phase. An effort was made to make the theme as concise and meaningful as possible. The coding including themes and sub-themes generation was conducted by all researchers. In total, six themes were generated. Each theme based on the data was explained explicitly. Considering the research question, these explanations were argumentatively presented and the most appropriate statements representing the theme were selected.

## Results

### Socio-demographic characteristics of the respondents

Table 1 summarizes the descriptive characteristics of the study participants. All the respondents were female ranging from 28 to 58 years of age. Six had an experience of 10–14 years. Four had postgraduate qualifications. Seven out of ten participants lived with family and three had completed postgraduate degrees. Three were unit in charges and seven were floor nurses. Three had completed masters degrees and four had tested positive for COVID-19 in the past while carrying out their duties in a maternity ward.

**Table 1:** Socio-demographic characteristics of participants.

Characteristics		Frequency
Age	**25–34**	**4**
	**35–44**	**3**
	**≥45**	**3**
**Sex**	**Female**	**10**
Work experience	**5–9**	**2**
	**10–14**	**6**
	**≥15**	**2**
Qualifications	**Vocational qualifications**	**3**
	**Graduate qualifications**	**3**
	**Postgraduate qualifications**	**4**
Lives with family	**Yes**	**7**
	**No**	**3**
Designation	**Floor Nurses**	**7**
	**Unit in charges**	**3**
COVID Status	**Ever positive (and recovered)**	**4**
	**Never positive**	**6**

### Findings of the in-depth interviews

**Fig 1:**
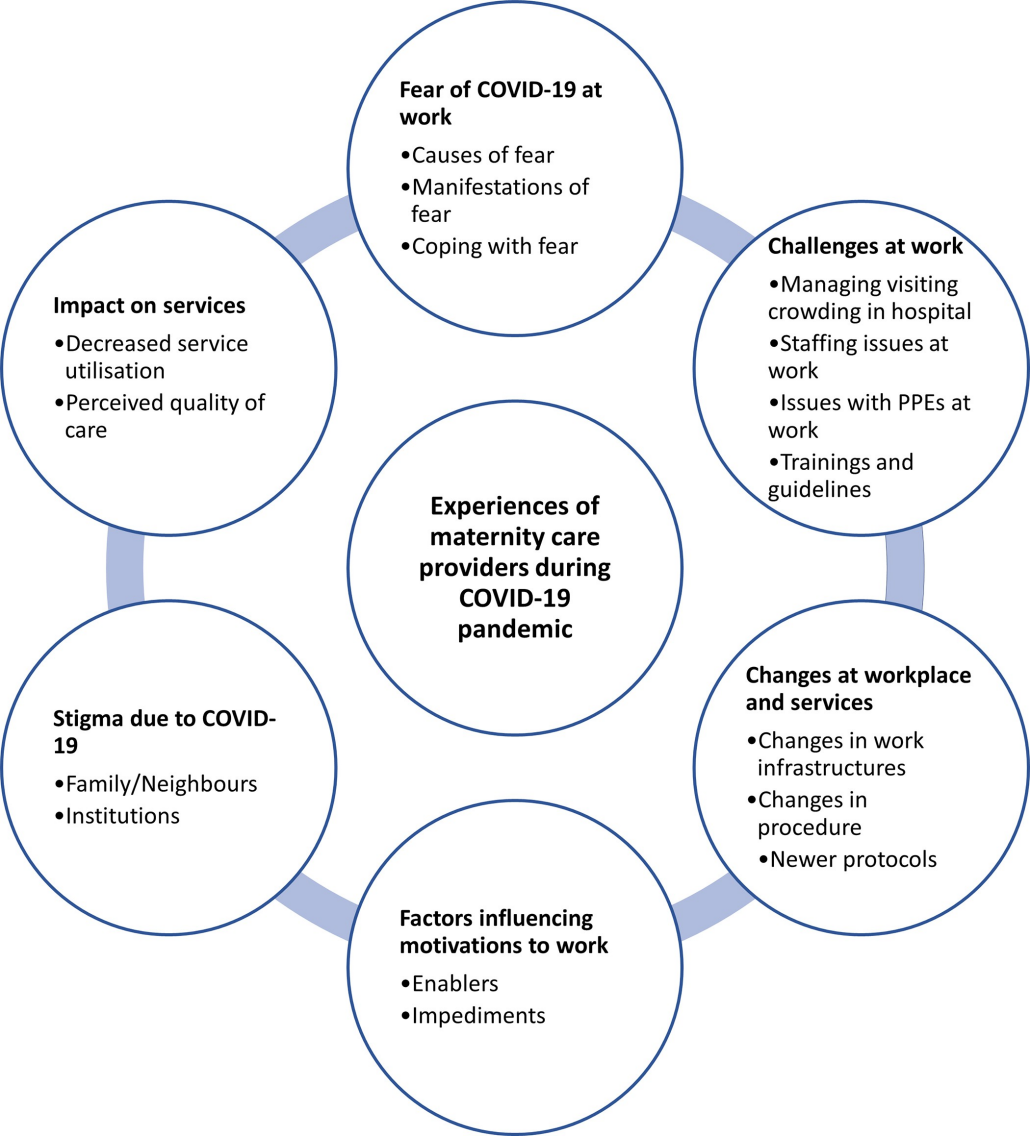
Themes and subthemes.

A summary of the themes and subthemes are presented in Fig 1.

#### Theme 1: Fear of COVID-19 at work

*Sub-theme 1*: *Causes of fear*. As mentioned by most participants, fear was perceived as greater in the initial phases when practices relating to COVID-19, its transmission rate and overall outcomes were uncertain. This fear was heightened by the risk of spread to a family member, in particular to the young and elderly groups. The fear was more prominent among health workers reporting a diagnosed chronic disease as well as those with family members reporting such conditions. Being ostracized from society was a common fear reported by several participants. It was also reported that much hearing potentially inconsistent or incorrect news from different media portals led to increased fear rather than allaying it.

*“… as my contemporaries started testing positive for COVID … the uncertainty around COVID further instilled more fear in me. … Later when I got posted in an isolation ward and saw many patients getting discharged. This allayed my fear to some extent…” P3*

*Sub-theme 2*: *Manifestations of fear*. Fear manifested in the form of anxiety ranging from being anxious at work, continuous thoughts of family members, irritability, loss of sleep, headache, excessive handwashing; both at work and home, and weight loss. A single participant complained of shortness of breath, dry cough, feeling feverish right after she came to know of the positivity of one of her patients. She later attributed these symptoms to a panic attack.

*“…I started washing hands frequently … I had repetitive thoughts of washing my hands even during sleep …” P1*

*Sub-theme 3*: *Coping with fear*. The majority of the participants reported waning fear as time passed. Being posted to COVID-19 wards and isolation made them more aware and knowledgeable about the nature of the disease, on ways to avoid transmission by wearing personal protective equipment (PPE), frequent handwashing, and the consequences of infection. They reported the gradual realization that testing positive for COVID-19 was not akin to death. The protracted nature of the COVID-19 pandemic resulted in some participants adapting downwards their perception of the virus’ risks, and placing lesser demands on quarantine and isolation.

*“I feared going near the [patient’s] bed initially, but now my fear has slowly decreased after being posted to COVID hospital.” P9*

#### Theme 2: Challenges at work

Under this theme, four sub-themes were generated as managing visitor crowding, staffing issues at work, personal protective equipment / measures, and training and guidelines.

*Sub-theme 1*: *Managing Visitor crowding in hospital*. Visitor control was criticized by most participants. The number of security guards was perceived as inadequate despite increasing their number. Participants reported a lack of coordination between the security guards and the visitors for managing behaviour within the health care setting. Participants reported visitors having misbehaved, and threatened staff even resorting to spitting on them. Respondents described a lack of sensitivity among visitors. One COVID-19 patient and their visitors absconded when being referred to the provincial COVID-19 hospital. The case could not be traced further. Respondents described that while soap, water and hand sanitiser were kept for patients’ attendants and visitors, the level of adherence to this type of infection control measure was unclear. Participants also criticized the low level of pandemic health literacy among visitors.

*“Managing extra people visiting the hospital was a real challenge for us. The number of security personnel was increased. This too did not work as the visitors verbally abused the security personnel and threatened to physically assault the personnel if they attempted to stop the visitors from entering the hospital. Furthermore, they spat all over the place when they were stopped. We try to do our best to minimize the number of visitors and motivate the visitors to comply with the hygiene measures. However, the compliance was poor as it seemed the visitors did not take COVID seriously so we could do nothing**.”** P2*

*Sub-theme 2*: *Staffing issues at work*. Participants described that problems relating to Human Resource management were prevalent before the pandemic. Yet this challenge was exacerbated by the pandemic as staff isolated or switched to rotational duties across the wider hospital.

Participants commented on the continued demands placed on the maternity service throughout the pandemic. Yet the pressure on services increased as referrals from private hospitals and peripheral health centres increasingly referred patients to this hospital for even minor interventions such as measurement of blood pressure. Against these odds, participants reported attempting to cope as well as possible.

Respondents highlighted that, like the rest of the hospital, maternity services were stretched before and during the pandemic’s impact. Staff redeployment was cited as being particularly challenging, with already-stretched staffing spread even thinner as health professionals strived to meet the population’s needs. Staff leave was cancelled, and one respondent reported the impact on her home life as she felt it increasingly difficult to provide care for her baby:

*“Whenever I talked with my neighbour, they advised me to take annual leave to stay home and take care of my child. However, being a government health worker, I was not allowed to take any type of leave during this period. This was so stressful for me to cope with.” P2*

One participant blamed the workload that prevented her from handwashing. She openly blamed this as the cause for her contracting COVID-19 in due process.

*One day I was in close contact with a patient, …providing cold sponging to a pregnant lady with a high fever. The ward was so busy that I could not find time to adequately wash my hands. Soon after that day, I tested positive for COVID. P1*

One of the participants, who works as a clinical ward manager, explained how it was a challenge for her to manage her duty roster because of the uncertainties introduced by the requirement to isolate staff who may have come in contact with the COVID patients. The pressure on staff was such that one respondent reported a colleague being asked to come to work despite being advised to isolate.

*“Although non-COVID wards have lesser patient flow, it is impossible to pool staff because our hospital has always had a chronic shortage of staff. In situations where pooling may be possible, the staff are reluctant to take up duties as they lack skills required for maternity services.” P6*

Later on, the provincial government supported our COVID-19 hospital by transferring school health nurses. This helped maternity staff by relieving them of some of their COVID-19 duties.

*Sub-theme 3*: *Issues with Personal Protective measures/Equipment*. According to most of the participants, an acute shortage of PPE was faced by the staff in the initial pandemic phase. The staff even went on strike to demand the PPE. Some staff reported self-purchasing their PPE which placed an additional financial burden on them and their families.

*“As most people lost jobs, many hospital staff were the only bread earners of their family. in addition, as the Hospital did not provide adequate masks, we had to spend our own money to purchase the masks at extortionist prices to protect ourselves. Even if the hospital provided salary on time would be great motivation to me and my staff.” P2*

The perception of a managerial lapse in the hospital was prominent in respondents’ testimonies. Respondents appeared unsure about where to request supplies for PPE and PCR test kits for the hospital. This confusion was a result of the recent changes in the health system and central government structures.

*“Our demand for PPE took long to go up the bureaucratic channel. When it did reach the right section of the hospital they were not clear about the procurement system in emergencies like the pandemic due to a lack of clarity of the administrative and financial regulations. Local philanthropic agencies finally donated some PPE to us” P8*

One participant coordinated with non-governmental organizations for the supply of PPE which helped to manage the PPE to deliver the service.

Some participants admitted that they felt it tedious to wear a mask at all times during the pandemic. They reported their unease with clinical situations, such as in the operating room, where physical distancing became impossible. They described that adhering to infection control standards at times was motivationally challenging. Participants also reported problems in accessing PCR testing at night (when required for emergency cesarean section).

*“It is not possible for someone to wear a mask while suffering from running nose and cough. When I was isolating at home as I was tested positive for COVID, it was so uncomfortable and suffocating.” P1*

*Sub-theme 4*: *Training and guidelines*. Despite the pandemic, four out of ten participants described the training as inadequate and inefficient. The training was only provided for infection prevention in general. Individual case management scenario discussions were not presented, making clinical interactions at times a challenge. Inconsistencies in testing guidelines were also noted between the hospital and local government bodies as a cause of confusion among many participants.

*“Prior to taking up duties in COVID hospital, we were only given general training on infection prevention at health care settings. However, we did not receive any guidance on how to approach and provide care to patients with COVID in the Intensive Care Units. Although I was not required to take up duties at COVID hospital as I was still breastfeeding my child, my staff were required to take up duties at COVID hospital without adequate training.” P2*

*“The policy for return to work after isolation differed between local and national government in terms of the number of days. So it was hard for us to decide whether to return after 2 weeks or 3 weeks.” P1*

The services provided to patients suffering from non-COVID-19 problems were noted suffering a great deal during the crisis. Separate operating theatres (OT) for COVID-19 and non-COVID-19 patients could not be provided. There were no proper guidelines to isolate maternal cases. Test results were returned 2–3 days after testing, which led to unnecessary exposure to other patients and staff. Participants described how these exposures were potentially avoidable, but such guidelines were lacking. One of the participants reported how a suspected patient had to be isolated in a separate paying room which prevented her from accessing the facilities of the safe motherhood program. There were additional financial consequences for the family arising from this.

*“…either having a separate operating room dedicated for COVID positive patients or operating on COVID positive patients at the separate COVID hospital would help reduce the exposure COVID amongst the staff.” P4*

*“The results for PCR test in our hospital takes up to 7 days. This creates an additional burden for patients who are admitted on a separate bed just to rule out COVID infection as the bed charges are ~10 USD/day. A patient recently came out negative for COVID who spent 7 days at the hospital was unable to pay the hospital charges of ~ 45 USD. As all expenses are out-of-pocket, this is just so unfair to poor patients who have little means to afford it. Lack of adequate communication by staff and unclear administrative/finance regulations on the provision of free beds has led to this mishap.” P6*

One of the participants reported that the arrangement of a dedicated COVID-19 active response team would be of great help in responding to the cases. Similarly, one other expressed that management of a dead body was not conducted appropriately. They went on to highlight those basic cultural and religious considerations mean a lot to patients and their families.

#### Theme 3: Changes at workplace and services

*Subtheme 1*: *Changes in workplace*. Participants reported that changes to the clinical ward environment were made: such as handwashing corners for patients and their visitors. Attempts to increase physical distancing were also noted, as the inter-bed space was increased and some beds removed. But this necessity to prevent transmission impacted capacity. But all of these changes were hampered by staffing shortages, as a result of isolation and quarantine requirements. To mitigate the staff shortages, some wards and the staff were merged. This resulted in additional pressure on staff to cope with increased workloads.

*Sub-theme 2*: *Change in procedures*. Admission criteria and induction criteria were modified. Elective gynaecological and obstetric procedures were postponed to accommodate for the increasing maternity workload and also to decrease the chances of possible asymptomatic COVID-19 transmission.

Participants reported attempting to adhere to PPE guidance. Patients along with their attendants were encouraged to wear masks at all times. Fetoscopes were replaced by fetal Doppler which made it easier and more reliable for monitoring and also reduced the frequency of antenatal visits. To further reduce the need for frequent visits to the patients, the participants attempted to consolidate their activities to reduce the number of separate interactions with patients. Dressing of patients was conducted at the bedside instead of a dressing room, while counselling of the patient and their attendant was conducted during this dressing period to avoid additional interaction.

Participants described their attempts to improve the health literacy of the patient’s attendants–recognizing the attendants’ role in reducing the number of interactions needed from clinical staff. Attendants were informally trained to provide low-level support such as discontinuing intravenous fluids, disconnections of fluids and providing minor nursing care.

Participants mentioned that their hospital had not initiated any telemedicine services but had provided uninterrupted maternity care services.

#### Theme 4: Factors influencing motivations to work

*Sub-theme 1*: *Enablers*. Participants described their motivation as arising from their professional responsibility to society. They reported valuing the support they received from family, peers and nursing managers (termed “in-charge”). The announcement of incentives from the government was also welcomed and described as motivating.

*“I had undue pressure from my family to quit my job due to fear of COVID. My line manager provided a lot of support for my mental health and welfare. This gave me confidence to convince my family and continue my job.” P7*

*Sub-theme 2*: *Impediments*. Yet some described experiencing no support and motivation from family and colleagues. While most participants criticized the news media whom they described as negative and de-motivating.

*There was also a negative influence of media on me. We were also on the frontline but only doctors’ work was recognized. This was frustrating and brought out dissatisfaction in me. P8*

*“Talking to my family and friends helped me a lot to overcome my mental stress. I had undue pressure from all of them to leave the job as they feared for my well being. To my disappointment, my manager also advised me to quit the job citing my age. This was hard for me to handle as I have always found pride in my work providing service as a health care provider” P8*

#### Theme 5: Stigma due to COVID-19

*Sub-theme 1*: *family and neighbours*. Most of the participants experienced social stigma with discrimination from the community, especially neighbours, while some faced harsh conditions even at home by their husbands, in-laws and near relatives. Some participants described how their children faced stigma among their peers by virtue of their mother working in a hospital. Some also faced stigma from their colleagues and the hospital administration.

*“My neighbours spread a rumour that I was COVID positive when I was home for 2 days. I felt stigmatized being labelled as COVID positive and people stared at me with suspicion and also ran away from me on the street. COVID has been used as a reason to stigmatise health workers. However many weeks later when one of them got infected with COVID and they needed my help. They started treating me nicely.” P8*

*“The discrimination towards health workers is so strong that they consider all health workers as a vehicle for COVID transmission in the community. Even my sister-in-law stopped talking to me. My children were not allowed to play in the public playground which is just in front of my house. This was hard for me to take on as my relatives were discriminating me, let alone the community people.” P7*

*Sub-theme 2*: *Institution*. One participant broke down sharing her experience. She said how a tree had fallen on her hospital quarters and it was difficult to cut it down and then repair the nearby pipework. She described a failure of administrators and neighbours to help and reports having gone without running water for three days.

#### Theme 6: Impact of COVID-19 at work

*Sub-theme 1 Decreased service utilization*. Despite maternal health services being comparatively less affected by the pandemic than some other parts of the hospital, the health-seeking behaviour of patients was commented upon. Patients elected to deliver at home, in particular during the early stages of the pandemic. They only presented to hospital services at the point when complications had arisen. Participants attributed several maternal and neonatal deaths to these behaviours. It was felt that this was compounded by the reduction in antenatal care (ANC) visits, which resulted in lower detection of late-stage pregnancy complications.

*“… we have observed increased fresh and macerated stillbirth … this may be due to lack of transportation for timely arrival to the hospital, late admission of women at 41 to 42 weeks of pregnancy, and decreased antenatal visits. We could have saved more babies had they arrived earlier in their pregnancy. Issues like lack of neonatal intensive care units, lockdown / restrictions limiting the availability of hospital transport and human resources crisis have impacted patient outcomes at our hospital.” P9*

One of the participants recalled the first maternal death attributed to the COVID-19 crisis. A pregnant female with twin pregnancies was referred to the hospital after four hours of delivery of the first baby when she could not deliver the second. On arrival at the hospital, it was already too late and she had a cardiac arrest. Cardiopulmonary resuscitation was initially successful, but both the second baby and mother eventually died.

Participants reported patients were reluctant to occupy the general ward beds and an isolated cabin was being demanded more frequently to avoid cross-contamination. This added to the workload pressure on staff attending to the cabin. It was also apparent that personal protective measures such as masks and gloves were less frequently used in the isolated cabins which undermined the very purpose of isolation in the first place. Due to problems coordinating across the service, suspected positive or confirmed positive mothers supported in the cabin were not able to easily access the safe-motherhood facilities.

*Patients prefer to pay more for staying in a cabin [Single room with attached bathroom] due to fear of COVID transmission in general wards. This has increased the workload in these cabins where there are always fewer staff. On the contrary, patients and visitors do not wear masks and the cabin rooms are always crowded with a lot of people visiting the patients. This is unsafe for everyone” P5*

*Sub-theme 2*: *Perceived quality of care*. A majority of the participants had experienced adverse impacts of the pandemic on care quality. Several reported a decrease in nurse-patient time and its impact on delivering effective care. The regular rounds from medical practitioners became less frequent during the pandemic, it was also observed that the time allocated per bed for clinical decision making was shortened. The participants voiced their frustration in their inability to provide the highest quality maternity care possible.

*“Before COVID, we cared for our patients more closely with frequent conversations and patting on the back or holding hands to make them feel cared for was common. This was appreciated by the patient as well. Now due to the distancing rules, I feel we are providing inadequate mental health support to the patients in terms of them feeling adequately cared for.” P8*

*“…The on-call physicians are reluctant to attend calls immediately and in most cases, they come only when called many times. This was not the case before COVID. Back then we had very prompt visits. …” P3*

One of the participants also mentioned that there was reduced application of Kangaroo Mother Care (KMC) for pre-term deliveries, at the same time as pre-term and low birth weight deliveries were increasing. Counselling for family planning (such as postpartum intrauterine contraceptive device insertion) was also less consistent. Counselling in regards to the care of the mother and baby was also not practised routinely. Moreover, due to fear of contracting COVID-19, patients began demanding early discharge from the hospital as soon as possible after delivery.

*“Before the pandemic patients were keen to let them stay longer in hospital as they perceived better postnatal care at the hospital, but now they wish to get discharged as soon as they deliver which is also risky as the patient may not receive adequate postnatal care,”. P7*

## Discussion

Maternity care nurses faced a wide range of challenges during the delivery of maternity care in the COVID-19 pandemic. Participants reported fear, its manifestation and the way they adapted to fear. They also described their various challenges in terms of human resources, personal protective equipment and visitor management. They reported the stigma they had felt from their families, neighbours and their institution. They expressed that self-motivation was critical to them carrying on despite these challenges. Participants outlined a range of strategies they adopted to cope with the change and many felt guilty over the compromised quality of care and increased neonatal mortality. These findings are relevant to maternal service planners and decision-makers in low income settings where health system relies more on patient and family members for supportive care of the patients and the cost of services are mostly borne out of pocket.

The perception of fear during the pandemic has been attributed in studies outside Nepal to a lack of understanding of the disease [21], the possibility of contracting the disease to self and family, lack of equipment [10,34] and information given by media [35]. Our findings are consistent with these reports. The impacts of such fears range from modification in behaviour to symptoms of depression, insomnia, anxiety and distress [34]. A previous study in Nepal reported that fear of COVID transmission amongst health care workers was aggravated by the inadequate provision of PPE [36]. Health service providers in time learn to cope with these fears. Mitigating activities include training, gaining first-hand clinical experience, peer and manager support, and institutional psychological support [37]. At an institutional level, the pandemic has precipitated several changes including a reduction in the number of beds, reduction in the length of stay, deferral of non-urgent gynaecological services, reduction of labour inductions and restriction in routine ANC and PNC checkup [27]. In a study conducted to identify the impact of coronavirus in Europe [38] online or phone consultation telemedicine was adopted as a virtual model to continue care provision whereas in this study, participants also believe that practising this model would have been advantageous.

In this study, participants expressed challenges in managing visitors to the maternity unit which is consistent with the findings from other low and middle-income countries including India, Bangladesh, Bolivia and Syria [27]. There are reports that patients’ visitors contributed to violent physical attacks on health care staff.

Heavier workloads related to the insufficient staff due to redeployment of staff to newly built facilities was reported in China [21]. Our findings also align with reports that maternity care was affected in several countries due to a shortage of staff owing to to COVID-19 symptoms, self-isolation and inability to reach the workplace due to restrictions [27]. In this study, participants described their preference that student health professionals and their teachers could be drafted to support front-line care. Some countries including Australia had utilised students in contact tracing and screening while acknowledging the consequent risk to student safety and security [5].

Lack of PPE reported in our study resonates with the shared feeling of ‘fighting a battle without equipment’ as expressed by health workers working during Ebola outbreak [11]. Shortage of gloves, masks and aprons [27], placed financial burden on staff to purchase PPE at very high market prices [39]. Discomfort and difficulties wearing PPE for extended durations was reported from other places as well [27]. Participants in our study described their frustration of inadequate PPE provision, hampered further changes in the administrative authority of hospitals between federal and provincial government coinciding with the pandemic [24].

Although the majority of the participants in this study had felt the training provided to them was insufficient, some participants were happy with the training they received. In other studies, participants felt the training had improved their confidence and competence [10] whereas some felt insufficient advice, training and information for case management [40]. Another issue identified in our study was consistent with other reports: that work was complicated by rapidly changing guidelines [37].

In this study, there were various enabling and complicating factors that influenced the participants’ ability to work during the pandemic. In line with other work [37], our study has identified a strong sense of professional duty that has driven health workers to continue their work despite the circumstances. Participants in our study also voiced their frustration with a lack of institutional and governmental support [41], in a similar way to reports arising from the Ebola epidemic in Liberia [10]. Unlike in our study, health workers during the Ebola epidemic received psychological support from their institution [11].

Participants in this study shared their experience of stigma from a range of sources: including family, neighbours, colleagues and administrators. These findings are consistent with a recently published study conducted from Nepal which revealed considerable stigma across the health care workforce [42]. Another study from Nepal also reported health workers asked by landlords to vacate the house in fear of transmission of COVID from the health workers [36]. That study, carried out in Nepal shows the difficulties faced by healthcare workers in finding food and shelter [43].

Most of the participants in this study reported reduced antenatal visits, with women preferring home delivery and late presentation to the hospital in the event of complications arising. During maternal and perinatal death reviews they had found an increase in intrauterine fetal death and stillbirth. Participants also mentioned the cause of maternal death during the lockdown as the consequences of the pandemic situation. Their experience also mentioned an increase in anaemic mothers an increased incidence of low birth weight and reduced practice of Kangaroo Mother Care. These findings are consistent with the national survey carried out in the UK for assessing maternity service during COVID -19 pandemic where they reported a reduction in antenatal and postnatal appointments [44]. Similarly, a prospective observational single-centre study conducted also shows the main reason for the delay in health-seeking behaviour by pregnant women was fear of getting infected and that resulted in complications during pregnancies [23]. Findings from the study carried out in Nepal to assess the impact of the COVID-19 pandemic on health service utilization shows the pervasive impact of lockdown on essential health services such as maternity services and immunization [24]. Another prospective observational study conducted to assess the effect of COVID-19 pandemic response in intrapartum care, still, birth and neonatal mortality in Nepal shows a decrease in institutional birth, increase in low birth weight, institutional stillbirth and neonatal death [17].

The strength of this study is that it is based within a major tertiary-level hospital contemporaneously with the pandemic, and represents the impact of COVID-19 in a comparatively under-researched geographical and resource-constrained context. The themes that emerge are relevant to policymakers and administrators seeking to improve health system resilience in similar contexts. However, some of the issues, in particular those relating to culture and stigma, may be specific to the Nepalese context. Furthermore, the extent to which specific issues can be generalized is uncertain.

## Conclusion

The pandemic has exposed the fragility of health systems in the unprecedented, overwhelming and protracted context of the COVID-19 pandemic, and despite so many impediments and challenges, maternity providers have continued services albeit with adaptations and limitations. Challenges faced by care providers are compounded by issues at the personal, familial, societal, institutional and governance levels. The pandemic has brought to the surface, the challenges faced by maternity providers from inadequate administration and governance by provincial and federal authorities that were admittedly weak even before the pandemic. Future pandemic preparedness planning should consider findings from this study to improve and protect the working conditions for maternal and child health staff during future public health emergencies in Nepal and elsewhere.

## Supporting information

S1 Table Master table with thematically coded data. (DOCX)
