# Supplementary material for: Experiences of nurses providing maternity care in a public hospital during the COVID-19 pandemic in Nepal: A qualitative study
Source: PLOS Glob Public Health. 2022 May 5;2(5):e0000322. doi: 10.1371/journal.pgph.0000322 (PMC10021459; doi:10.1371/journal.pgph.0000322)
Supplement: S1 Table — (DOCX) [file pgph.0000322.s001.docx]

**S1 Table: Master table with thematically coded data**

| - *Theme: Fear of COVID-19 at work* | | |
| --- | --- | --- |
| *Sub-theme: Causes of Fear* | | |
| Codes | *Narration* | |
| Negative news from the media | *I think the media should broadcast positive news regarding preventive measures for COVID 19. But some Youtube channels broadcasted only negative news regarding COVID 19 which created fear in the public. Only rarely did media create positive news for awareness amongst us. P8* | |
| Friend testing positive | *When my friend started testing positive, I too feared a lot.P7* | |
| Uncertainty of COVID | *…Initially, I had no fear. But when my contemporaries started testing positive for COVID, I was very much scared. The uncertainty around COVID further instilled more fear in me…P3* | |
| Transmission to self and family | *I had fear in the beginning when everything about COVID was unknown. Fear of death from COVID was present in the initial stage with doubt of surrounding people having COVID, risk of spread and transmission from them to me and then to my family. P1* | |
| Transmission to three-month-old baby | *There was fear of transmission to my three-month-old baby. Due to fear, I spent sleepless nights, thinking about the baby and the family even while working at work. I was so disturbed that I used to be physically present in the hospital but my mind was always with my baby. P2* | |
| *Theme: Fear of COVID-19 at work* | | |
| *Sub-theme:* manifestations of fear | | |
| Codes | *Narration* | |
| Lost weight | *Due to fear of transmission, I had not slept for many days and I lost about 4-5 kg.…P8. When I got a common cold, I was tensed doubting it to be a COVID infection., At the initial stage of COVID, I could not sleep at night but nowadays I sleep well. P8* | |
| Could not sleep well | *Due to fear of contracting COVID, I could not sleep well. As I was an asthmatic patient, I thought that If I get COVID, it will take my life.P4* | |
| Panic attack | *I had a panic attack. Once while returning home after duty I doubted if the disease got transmitted to me. I then started feeling difficulty in breathing, a dry cough, and a feverish feeling in the forehead. It was all because I panicked a lot.P1* | |
| Repetitive thought of corona | *I started washing my hands frequently and even awoke at the night for hand washing. Also, I had repetitive thoughts of washing my hands even during sleep. Repetitive thoughts about corona started coming to my mind. P1* | |
| *Theme: Fear of COVID-19 at work* | | |
| *Sub-theme:* coping with fear | | |
| Codes | *Narration* | |
| Community transmission phase | *…As COVID entered the community transmission phase, I realized that I am not only the one to spread the disease. Then my fear slowly faded away. P4* | |
| Posting to COVID hospital | *….but slowly I began to realize that it is here to stay and may last for months. Later when I got posted in an isolation ward and saw many patients getting discharged, I realized that COVID can be cured. This allayed my fear to some extent. P3* | |
| *Theme: Challenges at work* | | |
| *Sub-theme:* Managing visiting crowding in hospital | | |
| Codes | *Narration* | |
| Guards not staying at the gate | *We have a challenge in controlling the visitor before the pandemic and is continuing. Guard does not stay at the gate continuously. We had raised this issue to the management too but it has not been addressed. One patient has at least 5 visitors and the ward is like a fish market. Visitors are controlled only during round and cleanliness time. P9* | |
| Guard misbehaved by visitors | *Visitor control was a real challenge for us. Security was increased. But when the guards were stringent in restricting the visitors, the visitors attempted to harm the guards physically, spit everywhere and threaten them. We try our best to control the visitor but possibly due to a lack of sensitiveness in public we could do nothing****.*** *P2* | |
| Staying among ten visitors | *We have to stay among 10 visitors and work, We are not able to control visitors. The situation is the same as before. We are only taking precautions by frequent handwashing and wearing a mask. P10* | |
| Absconding of COVID positive patients along with visitors | *We did not encounter any problem in the first case. But during the 2^nd^ case, we had a problem. In the evening shift, we have only one sister for duty so when she was doing her work, 2nd case along with the visitor ran away. We had called for an ambulance but before the ambulance arrived, they ran away. They were scared of going to COVID hospital. This case could not be traced. P66* | |
| Non-compliance to instructions | *There was no change in the hospital. I didn’t see visitors restricted to visit patients. There was soap and water for handwashing and availability of sanitiser but don’t know whether they did it or not. […] 6-7 visitors come to see one patient; they do not comply with our request. We tried to the full extent possible from our side P1* | |
| *Theme: Challenges at work* | | |
| *Sub-theme: Staffing issues at work* | | |
| Codes | *Narration* | |
| Increase case flow | *During lockdown from Chaitra to Baisakh, COVID cases decreased by 75% and then after people started feeling corona is nothing and thought nothing will happen by being scared and then cases increased during Shrawan and Bhadra. So, we can say that Case flow was increased in comparison to the previous stage due to the closing of other hospitals. P1* | |
|  | *We also took care of gynaecological outpatients. which created an increased workload. We are providing service to all pregnant mothers from periphery areas in these situations. A pregnant mother from the periphery comes even for minor procedures like taking blood pressure. During the lockdown, very few clients came for family planning services. P8* | |
| Continuation of service | *Maternity service does not stop in a pandemic. Many private hospitals stop giving maternity services during a pandemic. Being a government hospital, we cannot stop our service. P9* | |
|  | *Despite our staff being infected and our obligation to send staff to quarantine, our service was never interrupted. When our staff was positive, we fumigated our ward for a week and went to management for requesting to halt the service for a while but management denied it. So, despite difficulties, we continued giving service. P9* | |
| Allocation of maternity staff to COVID hospital  No posting of students, teachers and trainees | *We were told to send our staff to COVID hospital and isolation which affected our ward. Even I told our matron that I find it difficult to allocate our staff to COVID hospital as our caseload was still the same despite the lockdown. It was here that I felt we as nurses were not united. The case flow of other wards was less and staff were idle. It would have been best if they were sent to COVID hospital. If I send one staff to COVID hospital for 14 days I lost her for a month because further 14 days I have to send her into quarantine. Matron could not advocate in our favour in this matter. We could have sent our staff in this understanding that we would be getting support from other wards where the caseload was less but nobody cooperated with us and blamed us that we were one to receive maternity allowance and we should give the service. But later on, we again discussed the matter with the matron and the hospital director and our voice was finally heard and our staff were not sent to COVID hospital. P9*  *We also have to go to the provincial COVID hospital. We were already insufficient with no student, volunteer or trainee being posted. This further strained our service. But our service was not interrupted. We were obliged as private hospitals were in a closed phase and those hospitals that had been providing maternity service through the safe motherhood program had stopped giving the service. P2*  *During COVID, the difficulties we faced due to a lack of human resources were intensified. Previously, we had students, teachers, and trainees and the scarcity of human resources was somehow managed. But during COVID all nursing and paramedics college was closed. We had a maximum of 3 staff in each shift; one must manage the labour room, waiting room and admission and very difficult in giving quality care. We have started training and we have training participants now but is not continuous. So, the frequency of monitoring by staff was decreased and it was nearly impossible to complete the partograph charts. P9* | |
| Duties during quarantine days | *When my friend tested positive, we were quarantined in a hotel for seven days. Even in quarantine, we were obliged to be on duty. I was away from my child for seven days. P7* | |
| One staff to care for many | *I think we need more human resources during these periods, but there are limited human resources in our unit to manage it. 1 staff had to cater service to 50-60 mothers in a day.P8* | |
| Difficult to maintain a duty roster | *It was very difficult to go along with a duty roster because we never knew who is going to be positive. However, my staff were very much supportive and it became easier to manage even in a crisis. P2* | |
| No provision of leave | *Whenever I used to meet my neighbour, they used to tell me to take leave and care for my baby. Being a government health worker, I was not allowed to take any type of leave during this period. This further used to add my stress.P2* | |
| Increase workload | *There was a pregnant mother with a 103-degree fever during my duty. I did work like giving cold compression and caring for the mother having direct contact with her. Ward was so busy that I didn’t get time to even wash my hands. So, I got exposed due to this reason and I was COVID positive. P1* | |
| Recruit adequate human resources | *My main recommendation for management is to recruit adequate human resources. We don’t have even an alternative if one falls sick.P2* | |
| Pooling of staff from other wards | *Although patient flow is less in other wards like surgery pooling of staff can be a solution but it is impossible to pool staff because the human resource crisis is so much an endemic problem in this hospital. Even pooling is a challenge. the staff from other wards will start to tell they do not have maternity training. We do not have an incentive so why do we need to work there. P6* | |
| *Theme: Challenges at work* | | |
| *Sub-theme: Issues with personal protective* *equipment’s/measures at work* | | |
| Codes | *Narration* | |
| Insufficient PPE | *It would have been better if there was sufficient PPE and we could wear full PPE for each case. P4*  *For the initial 12 to 15 days of lockdown, there were not many cases. We were using a personal protective device like PPE, gown, gloves, and visor for checking those who came. Initially, our administration was haphazard so we did not get sanitiser but later we got. Previously case flow per month was 1250-1300 now still we are checking 900-950 per month. P8* | |
| Self-purchase of PPE | *We face a lot of problems and are still facing them. Initially, we were not supported by the hospital so were given any PPE. Due to the rumour of the spread of disease, we began to buy on our own and start wearing masks and gowns. But how can we manage every day? We had to dry and wash it. supply was then made available and now the situation is far better.p8* | |
| Support from organizations. | *I had feared it initially and we also had a crisis of PPE while delivering the service so I coordinated with IPAS (NGO) for PPE so it was easy for me to manage the crisis. Now I have solved the problem of PPE.P8* | |
| Dilemma on demanding PPE | *We went as per our chain of command to demand PPE but our higher authority was in a dilemma where should they get support. As our country is just entering into the federal system, our management was also confused in coordinating, corresponding and liaising for the supply of PPE and PCR testing. Later local agencies also supported PPE. P8* | |
| Interruption of water supply | *We did not compromise on providing quality care to the patients. But sometimes crisis of water to maintain hand hygiene and service may be delayed. P8* | |
| Difficult in maintaining personal protective measures | *I feel WHO has created unnecessary fear to prevail like how can someone wear a mask while suffering from a running nose and cough. I tried wearing a mask even in my room while I was COVID positive but it was so uncomfortable and I felt so difficult as if I will die from this difficulty. p1*  *Our patients are in labour pain and it is impossible for us to maintain physical distance and provide care. We have to check the progress of labour and check FHS frequently. Our area is very much sensitive. P7*  *We maintain social distance during our duty hours except for operation time when it was not possible. Patients undergoing OT are obliged to wear a mask but they complain of difficulty in breathing. P4*  *To date, we did not encounter any COVID positive cases for the caesarean section. But after completion of the case, one was found to be positive. For the planned case we do mostly PCR before the operative procedure. But for an emergency, it is not possible and in maternity, we mostly encounter emergency cases. P4*  *It is impossible to screen all maternity cases as they might come in an emergency and at night and we may have to intervene immediately. P9* | |
| *Theme: Challenges at work* | | |
| *Sub-theme: Training and guidelines* | | |
| Codes | *Narration* | |
| Ineffective training  Unable to participate | *There were no training sessions but I gathered information regarding COVID from different internet media like Facebook, youtube, etc. I heard the Nursing Association of Nepal had conducted a session on motivation but could not take a class. But I had taken half-hour class given by staffs but they didn’t teach us properly like as if they even don’t know. It might be because youtube was used so it wasn’t of enough quality as performing it wasn’t like we were taught. Moreover, our government don’t have a fixed guideline*  *P1* | |
| Insufficient content | *we did not receive on caring for a covid patient in ICU. I being a breastfeeding mother was not sent to covid hospital but my staff were. Donning and doffing of PPE Training was provided by the institution in a group of 5-10 but I don’t feel adequate in viral infection management. Moreover, I am also not aware of any maternity-related guidelines for managing mothers during covid. P2*  *Since it is a new disease, they should have given us orientation and training but we are not given to that extent. P4*  *There is no specific guideline for patient management neither we have received any orientation. All are doing their best on their own. P6* | |
| No provision of prompt result | *Patients get admitted to the ward and then they send the sample some will have a positive result. They are in contact with many wards so how much area should we do fumigation. You were asking me what new practices have been added during this pandemic this fumigation is one. But I don’t see effective. We are not sure with time some say it is 20 min some say 40 min, 1 hour. The fact is time depends on the space but no one knows for what space how much time is required. So, I left doing fumigation.P6* | |
| No fixed guideline for retesting | *I don’t like the rule of government that is we are pressurized for returning to work as there is a patient load in the hospital because some authorities advise repeating PCR in 15 days while the city council advise in 21 days. But the hospital says to come in the job after 14 days of staying in isolation for attendance.P1* | |
| No response teams  No proper management of dead body | *We have nearly 13 to 14 departments. It would have been better if the hospital could have prepared a response team. I don’t know whether there is such type of team or not and even if it is there they are just for namesake and incentive. P6*  *I think the dead body of the covid patient should be given to her family for see and do their cultural ceremony by certain persons of family members by wearing safety measures. The government have to make a guideline on this part. P8* | |
| *Theme: Changes at work and services* | | |
| *Sub-theme: changes in work infrastructures* | | |
| Codes | *Narration* | |
| Handwashing corner for visitors | *We made a handwashing corner for patients and visitors and started wearing PPE more effectively and tried to control the visitors to some extent. P5* | |
| Merging of ward | *There was a merging of the wards so that we were less exposed and the time exposed was minimized. P1* | |
| Reduce in bed  Did not allow visitors  Allocate separate room for delivery of COVID positive  Suspend routine cases | *We made many changes in our practice; we reduced beds to 2 from 3 and maintained a distance of 1.5 m in the admission room of the maternity ward. Initially, we kept the visitor outside the admission room and kept only the pregnant mother in the admission room. We removed the beds from the observation and waiting rooms and maintained a distance of 1 meter between the beds. If we encounter COVID positive pregnant mother then we had planned to deliver the mother in the MVA room as we have another labour table over there too. We had arranged in such a way that COVID positive pregnant mothers will not come in contact with other pregnant mothers. Immediately after delivery, we had planned to send the mother to the cabin instead of the postnatal ward. We had planned in this manner but to date, we have not encountered any such cases. We encountered a pregnant mother with fever, we sent PCR immediately and conducted her delivery with full precaution. The next day the result of PCR came positive and we disinfected the labour room. P9*  *. …..We remove 12 beds to adjust the distance of 4 feet but later on as it was impossible again bed was attached. P2* | |
| Increase workload by merging | *As staff started testing positive, our HOD decided to keep half of the staff at work and the other half were sent home to minimize the rate of infection without disrupting the service. For managing staff during this time, we merged the postnatal and cabin with the maternity ward. But this could not last longer than two weeks as there was work overload. There were 18-20 normal cases and 10-12 CS every day. Its visible impact was seen in quality care. It was difficult to monitor the mother and fetus. Then again, we decided to run our ward in a full-fledged manner. We are also planning to run the other gynaecological cases after the PCR result. P9*  *To minimize the workload and exposure. We merged our ward and formed a single ward. But that also did not last long because other hospitals did not take the inpatient cases and the caseload become high in our hospital, especially in the maternity department. There is an increase in the trend of the patient as well as staff being positive. We were obliged to manage duty by not taking a single day off. For this, I had to counsel some staff for duty for 1 to 2 hours. Staffs tell that they have taken care of the positive cases throughout the day and know-how can they go home so I had to arrange for accommodation here at the hospital. In that case, I have counselled them for duty by arranging them at a cabin. But to date, no one has stayed in the cabin they have arranged themselves at their home. P6*  *When our one staff was seen positive our ward was sealed. Later on to minimize exposure merging of the ward was done. But it was very difficult to manage the work. I even don’t have time to eat. P3* | |
| *Theme: Changes at work and services* | | |
| *Sub-theme: Changes in the procedure* | | |
| Codes | *Narration* | |
| Change in admission and discharge | *We changed the admission criteria of the mother. We assess all the pregnant who come to maternity admission and admit them based on the protocol we have re-designed. There was a little change in discharge procedure for a few months like discharging the mother after 6 hours which was 24 hours before the COVID pandemic.P1*  *We make patient wash their hand properly immediately after the admission procedure and change the bedsheet before keeping the patient in bed. At present we have sufficient PPE for caring for the patient but sometimes we lack masks and we buy on our own.P3* | |
| Suspend routine cases | *……All the routine gynaecology cases like TH, VH were suspended….P9* | |
| Use of effective Personal Protective Equipment (PPE) | *Previously we used to wear incomplete PPE haphazardly. But at present, we are wearing it appropriately and we are also forcing mothers to wear a mask. Other practices are as usual like delayed cord clamping, skin to skin contact and breastfeeding practices etc. even in the suspected cases. P9*  *There were some major changes in working place like we started wearing masks, and caps. We started using sanitiser and did frequent handwashing. P10* | |
| No induction | *During the peak of the pandemic, we did not conduct induction for 2 to 3 weeks then was conducted on a need basis. P9* | |
| Use of doppler | *Previously we did not use Doppler but nowadays we use Doppler for taking fetal heart sounds. P10*  *Previously we used to check fetal heart sounds by fetoscope. Now we had started taking by Doppler.P7* | |
| Shift from head to toe to focused examination | *We perform detailed head to toe examinations only for the needful ones otherwise we take history only. We focus on danger signs. I think we gave TT vaccine to all pregnant mothers in the Morang district. P8* | |
| Design of maternity card | *We had designed a new maternity card for assessing patients..P1*  *During the pandemic, we set the new admission criteria. And had also developed an emergency maternity card. Whoever comes to the emergency of the maternity ward we told them to have an emergency maternity card and record all the assessments we have done on that card. Whoever is in false labour we prescribe them analgesics and sent them home. This system has made our work easier. P9* | |
| Decrease nurse-patient time | *I felt different while dealing with patients Before COVID we talked with patients a lot and touch them to make them feel better. Nowadays we maintain physical distance while caring for the patient it makes me feel bad about our compromised nursing care. P8*  *We minimize the frequent visit to the bedside. Patient counselling was done but not as before. it was given during dressing at the bedside. All activities were carried on during the visit made to the patient side after wearing PPE. P2*  *Previously we used to come running to address the patient's problem. But now we think, assess and wear complete PPE and only go to see the patient. For minor tasks like closing the drip, we have taught visitors to do that. For taking medication and taking vitals signs only we go to the patient side by wearing PPE .P3* | |
| No telemedicine facilities | *Our hospital has not initiated any telemedicine services but our hospital is open and we are offering 24-hour services to whoever comes to the hospital. It is difficult to implement telemedicine, and it would increase our workload. P9* | |
| *Theme: Changes at work and services* | | |
| *Sub-theme: Newer protocols* | | |
| Codes | *Narration* | |
| Change in admission and discharge | *We changed the admission criteria of the mother. We assess all the mother who comes to maternity admission and admits mother based on the protocol we have re-designed. There was a little change in discharge procedure for a few months like discharging the mother after 6 hours which was 24 hours before COVID.P1*  *We make patient wash their hand properly immediately after the admission procedure and change the bedsheet before keeping the patient in bed. At present we have sufficient PPE for caring for the patient but sometimes we lack masks and we buy on our own.P3* | |
| Suspend routine cases | *……All the routine gynaecology cases like TH, VH were suspended….P9* | |
| Use of effective Personal Protective Equipment (PPE) | *Previously we used to wear incomplete PPE haphazardly. But at present, we are wearing it appropriately and we are also forcing mothers to wear a mask. Other practices are as usual like delayed cord clamping, skin to skin contact and breastfeeding practices etc. even in the suspected cases. P9*  *There were some major changes in working place like we started wearing masks, and caps. We started using sanitiser and did frequent handwashing. P10* | |
| No induction | *During the peak of the pandemic, we did not conduct induction for 2 to 3 weeks then was conducted on a need basis. P9* | |
| Use of doppler | *Previously we did not use Doppler but nowadays we use Doppler for taking fetal heart sounds. P10*  *Previously we used to check fetal heart sounds by fetoscope. Now we had started taking by Doppler.P7* | |
| Shift from head to toe to focused examination | *We perform detailed head to toe examinations only for the needful ones otherwise we take history only. We focus on danger signs. I think we gave TT vaccine to all pregnant mothers in the Morang district. P8* | |
| Design of maternity card | *We had designed a new maternity card for assessing patients..P1*  *During the pandemic, we set the new admission criteria. And had also developed an emergency maternity card. Whoever comes to the emergency of the maternity ward we told them to have an emergency maternity card and record all the assessments we have done on that card. Whoever is in false labour we prescribe them analgesics and sent them home. This system has made our work easier. P9* | |
| Decrease nurse-patient time | *I felt different while dealing with patients Before COVID we talked with patients a lot and touch them to make them feel better. Nowadays we maintain physical distance while caring for the patient it makes me feel bad about our compromised nursing care. P8*  *We minimize the frequent visit to the bedside. Patient counselling was done but not as before. it was given during dressing at the bedside. All activities were carried on during the visit made to the patient side after wearing PPE. P2*  *Previously we used to come running to address the patient's problem. But now we think, assess and wear complete PPE and only go to see the patient. For minor tasks like closing the drip, we have taught visitors to do that. For taking medication and taking vitals signs only we go to the patient side by wearing PPE .P3* | |
| No telemedicine facilities | *Our hospital has not initiated any telemedicine services but our hospital is open and we are offering 24-hour services to whoever comes to the hospital. It is difficult to implement telemedicine, and it would increase our workload. P9* | |
| *Theme: Factors influencing motivations to work* | | |
| *Sub-theme: Enablers* | | |
| Codes | *Narration* | |
| Motivating measures | *To motivate me, I had left listening to the news. I spent the remaining time with my baby so I feel fresh in doing my duty. P2* | |
| Self-motivation | *Motivation should come from the inner self, I am a nurse, and I have read about this subject. Willpower must come from within ourselves. So it must come from within myself in this challenging situation, I have read about this subject, it is my profession, And so I should serve support at this time to my country, family and society, as a health professional. I shouldn’t run away from my duty. P1* | |
|  | *I don’t feel bored doing my duty. I did work for this long and now I am near retirement. I feel like doing work for a longer period. P10* | |
|  | *I am near retirement and wanted to serve more and more. My instinct drives and motivates me to do work in these situations because I am a nurse. P3* | |
| Support from daughter | *My daughter is married and staying at her home. She is also a nurse. She came to my home during COVID and advised me of many precautionary measures. Whereas my son told me to take leave and not to go to the hospital. But I can not do that. P3* | |
| Staff as strength | *Being in charge, I faced both technical as well as managerial problems. Many were in the condition to leave the job, some even left. Even for me, my family and in-laws told me not to go to my job and take a month's leave. But this was not a solution. I have a responsibility to make others also do the work. But the good thing is my staff always supported me. During a crisis, they even did double duty and manage. In this way receiving support from my staff boost me to work. Later on, my family also realize and support me. P6*  *We both husband and wife work out of the home. We are not sure how did we become infected. Besides all this, I have a supportive family. No one in my family blamed me. During my positive, my colleague in charge was in regular contact with me. This was great motivation for me. P4*  *The ward in charge and my colleague supported me a lot. My in-laws and husband pressure me to leave my job. I was on the verge to resign but my in charge counselled and supported me a lot. This gave me the confidence to continue my job. P7* | |
| Recognized by media | *The media has shown positive as well as negative responses. But I had taken news from the media positively as it has recognized us as frontlines. They are recognizing not only the work of doctors but also of nurses. P6* | |
| Announcement of allowance | *Rather than our Hospital, I feel that our government has motivated us. The announcement of giving an extra allowance is great motivation for us but we have received it only till Chaitra (March/April). P2* | |
| *Theme: Factors influencing motivations to work* | | |
| *Sub-theme: Impediments* | | |
| Codes | *Narration* | |
| No counselling from the hospital | *Hospital had done nothing to support positive staff. In my opinion, the hospital should do counselling for all staff regarding pre and post covid complications and problems. I think this counselling helps clients to do immediate follow-up and care if a problem crisis. P3* | |
| No support from media and society | *I had no motivation from society and even from the media negative news was given. So, even from negative news, we have motivation p1*  *There was also a negative influence of media on me. We were also one to work as frontline but only doctors' work was recognized. This was really frustrating and brought dissatisfaction to me. P8* | |
| No support from family | *..I feel like resigning from my job as I had a 2-year-old baby. Family members were also forcing me to quit the job. I resisted and they told me to leave the house and lived separately. They did not take proper care of my baby as well. I realized that it would not be wise to give pressure and stress on my in-laws who were also heart patients. So, I left my in-law’s house and lived separately in a rent. I, my husband and our child are now living in a rented house...” P7* | |
| Uncertainty about receiving allowances | *We have nothing, not even words of that you do work we will do this task for you. The government had said about giving hazard allowance but we still have not received it. I have received the only incentive for working in COVID hospital, no other incentive as said by the government. P1* | |
| *Theme: Stigma due to COVID-19* | | |
| *Sub-theme: Family/Neighbours* | | |
| Codes | *Narration* | |
| Not allowed children to play | *My sister-in-law is just next to my house but she did not allow my children to enter her house due to fear of transmission. She stopped talking to me. P7* | |
| Not visited | *Even when I become negative nobody even my close son and daughter in law didn’t visit my house. P3* | |
| Not allow children to play | *In front of my house, there is a big playground, my child used to go there for playing. Since I used to work in a hospital, community people warned me not to send my children there. This hurt me a lot. I counselled myself that if my family are behaving in this manner then what can I expect from other people. P7* | |
| Bitter experience | *I had a bitter experience with my neighbour. They did not even talk to me. Such a response demotivated me a lot……P3* | |
| Spreading of rumour | *My neighbours were not good to me. When I did not go to the office for two days they spread the rumour that I got infected. Whenever I met them on the way even when I and they both were wearing masks, they used to cover their face with their hands. I really used to feel bad when children at my neighbours run away when they saw me. We were living so closely with our neighbour but all of sudden they were distancing from us. P8* | |
| Unsupportive behaviour | *…. due to pressure from my in-laws, I left my home and I have to suffer a lot in finding the rent. As I am a health personnel, I was denied a rental flat by the owners. P7*  *Due to construction going on at my house, I used to keep my motorbike at my neighbour's house. They told me either to keep it there permanently or take it away but not to come frequently. So, I started keeping at another house that supported me………..During my posting at COVID hospital, I being in charge was posted in the green zone and was planning to manage from my home but my husband told me to stay at a hotel arranged by the hospital due to fear of being stigmatized by the society, P5*  *Our next-door neighbour got angry with us. They turned their face whenever they saw us. They even stopped communicating with us. Nobody in this society asks us about our condition. Our basic groceries were brought to us by our relatives but people in this society did not ask us anything.P4*  *I had a bad feeling of unsupportive behaviour experience from neighbours staying in the quarter. My front neighbour treated me in such a way as if COVID will transmit even by speaking. Even intelligent people are treated as if corona will spread by speaking. I was told by a neighbour to close my doors and windows and even not to come outside of my room to dry my clothes outside P1* | |
| Not visited by relatives | *After I left my job at BPKIHS, Dharan, (my previous job) me and my husband decided not to stay in the quarter and go to my home in Bargachi (local town). Even though we were negative, society people did not come to our house. When we stay on the balcony, surrounding people told us why we were not wearing a mask. Then we return to our quarter again.P10*  *I had a bitter experience with my neighbour. Even when I become negative nobody even my close son and daughter in law did not visit my house and neighbours did not even talk to me. Society’s response stressed me a lot. P3* | |
| *Theme: Stigma due to COVID-19* | | |
| *Sub-theme: Institution* | | |
| Codes | *Narration* | |
| Placed register outside | *I have no motivation from the hospital management team. They have not even conducted a meeting. People from the hospital administration treated us inappropriately. They placed the register outside so that we could be separated from them. Neither measures for visitor control nor adequate sanitisers are present. We re-use gloves by boiling. Even when complaining about the torn gloves, we didn’t get the supply of new sterilized gloves from the hospital supply section. P1* | |
| No repair of waterpipe | *..I had many problems, a big tree fell and hit my room ceiling. With so much difficulty and so late, the tree cutter came for its solution and then again pipeline of the tap was cut down in this sequence. I asked for help from neighbours after I had no water supply at last when only some portion was made by maintenance but again didn’t come the other day leaving the task incomplete. With much pleading effort, I phoned management for help and sir from administration finally brought a new pipe. Till this difficult time, no neighbours for 3 days from quarter never even asked anything. I didn’t even bathe for 3 days due to this problem. Even the shopkeeper deny bringing water to my place. (sobbing) P1* | |
| Untouchability even after a negative result | *I did not get support from the hospital administration. They treated me very badly. When I went to admin for submitting my sheet role, they told me they will not touch the paper touched by me. P3* | |
| *Theme: Impact on Services* | | |
| *Sub-theme: Decreased service utilisation* | | |
| Codes | *Narration* | |
| Increase stillbirth,  No ANC visit | | *We conduct a maternal perinatal death review every month and it shows that we have 11 fresh and macerated stillbirths in Shrawan and Bhadra which may be due to no transportation and late admission of the mother at 41 to 42 weeks, no ANC visit. We could have saved many fresh stillbirths and low birth weight babies. Lack of NICU facilities, lockdown and human resources crisis we could not monitor patients well and save their life.P9* |
| Increase premature, low birth weight and IUFD,  Decrease ANC visit | | *I am observing that there is an increase in intrauterine fetal death, premature delivery, low-birth-weight ranging from 2-to 2.5 and anaemic mothers. Low birth weight may be a lack of nutritious diet, many lost their job during the lockdown, maybe they don’t have money to buy food; many could not do an antenatal checkup. When I checked the card, I saw that many had not completed four visits. P10* |
| Delay in receiving care | | *I am feeling that IUFD cases are increased. This may be because of our new admission criteria of 41 weeks. We have told them to come early if they have any problems but even, if they have a problem they come late. I also feel that ANC visits are also decreased and this may be due to the unavailability of transportation. P7* |
|  |  | *One case came 15-day post-dated. They had early neonatal death and that baby was precious to them. Due to fear of transmission, they did not come to the hospital. She felt difficult and her mother in law brought her here, there was IUFD, and the baby was expelled through CS. Mothers survive in this case.P6*  *To date after lockdown, we had one maternal mortality which was a case of twin delivery from Siraha. She had delivered one baby at her own place and could not deliver the other and was referred 4 hours after the delivery of the first. The first twin was delivered at 11 am and the mother was brought at 5 pm. It was 6:30 am when the Admission process and investigation were completed. Till then mother was in gasping and was revived after CPR and OT were done. After OT was kept on a ventilator. After 12 hours in ICU, she expired. I felt that partly lockdown is the cause of her death and partly it was their carelessness. P2* |
|  | | *Once Doctor kept a suspected case in a 105-bed number. After patients are kept here they have to pay 1200 per day. It took 7 days for the PCR report to come. If we have told as urgent it would have been quicker but we did not know. Although the report came negative he was not able to pay 5000 Nepali Rupees. This is a mismatch, if there was proper coordination he would get free delivery service under the safe motherhood program.* |
| *Theme: Impact on Services* | | |
| *Sub-theme: Perceived Quality of care* | | |
| Codes | *Narration* | |
| Difficult to give minimum care | | *There is no quality care. Even if we could give minimum care that was considered an achievement. How can we expect quality care from one staff? We don’t have any fixed guidelines we are just working on trial and error. P6* |
| No kangaroo mother care facility | | *We have a separate KMC room and used to give KMC to a low-birth-weight baby. But during the pandemic, we are not doing it. We are just counselling for skin to skin contact for low-birth-weight babies. P2* |
| Fever screening first priority | | *Previously we used to counsel more on the care of mother and baby after delivery. But nowadays initially we ask whether she has a fever or any symptoms of COVID or not and do accordingly. P7* |
| Reduce counselling | | *There was a change, Due to fear of contracting the disease, no counselling was provided. P1*  *Previously we used to counsel on PPIUCD very effectively but nowadays it has reduced because there is fear of transmission in direct communication and we have insufficient human resources too. P9* |
| Decrease nurse-patient time | | *I felt different while dealing with patients Before COVID we talked with patients a lot and touch them to make them feel better. Nowadays we maintain physical distance while caring for the patient it makes me feel bad about our compromised nursing care. P8*  *We minimize the frequent visit to the bedside. Patient counselling was done but not as before. it was given during dressing at the bedside. All activities were carried on during the visit made to the patient side after wearing PPE. P2*  *Previously we used to come running to address the patient's problem. But now we think, assess and wear complete PPE and only go to see the patient. For minor tasks like closing the drip, we have taught visitors to do that. For taking medication and taking vitals signs only we go to the patient side after wearing PPE .P3* |
